# Microfluidic Biochip Integrated with Composite Gel Composed of Silver Nanostructure @ Polydopamine–co–Chitosan for Rapid Detection of Airborne Bacteria

**DOI:** 10.3390/bios15110720

**Published:** 2025-10-30

**Authors:** Xi Su, Xinyu He, Chuang Ge, Yipei Wang, Yi Xu

**Affiliations:** 1 School of Advanced Materials Engineering, Jiaxing Nanhu University, Jiaxing 314001, China; 2 Key Disciplines Lab of Novel Micro-Nano Devices and System Technology, Key Laboratory of Optoelectronic Technology and Systems, Ministry of Education, Chongqing University, Chongqing 400044, China; 3 College of Chemistry and Chemical Engineering, Chongqing University of Science and Technology, Chongqing 401331, China; 4 Key Laboratory of Translational Research for Cancer Metastasis and Individualized Treatment, Chongqing University Cancer Hospital, Chongqing 400030, China; 5 School of Optoelectronic Engineering, Chongqing University, Chongqing 400044, China

**Keywords:** detection of airborne bacteria, microfluidic biochip, surface-enhanced Raman spectroscopy (SERS), silver nanostructure @ polydopamine–co–chitosan composite gel, bacterial capture and enrichment

## Abstract

Rapid detection and identification of airborne bacteria are critical for safeguarding human health, yet current technologies remain inadequate. To address this gap, we developed a multifunctional biochip that synergistically integrated a heptagonal micropillar array with a silver nanostructure–polydopamine–co–chitosan (AgNS@PDA–co–CS) composite gel to achieve highly efficient sampling, capture, enrichment, and in situ SERS detection of airborne bacteria. The integrated micropillar array increased the capture efficiency of *S. aureus* in aerosols from 11.4% (with a flat chip) to 86.3%, owing to its high specific surface area and its ability to generate chaotic vortices that promote bacterial impaction. Subsequent functionalization with the AgNS@PDA–co–CS gel improved the capture efficiency further to >99.9%, due to the synergistic effect of the gel’s adhesive properties and the abundant capture sites provided by the nanostructure, which collectively ensure robust bacterial retention. The incorporated AgNS also served as SERS-active sites, enabling direct identification of captured *S. aureus* at concentrations as low as 10^5^ CFU m^−3^ after 20 min of sampling. Furthermore, the platform successfully distinguished among three common bacterial species—*S. aureus*, *E. coli*, and *Bacillus cereus*—based on their SERS spectral profiles combined with principal component analysis (PCA). This work presents a synergistic strategy for simultaneous bacterial sampling, capture, enrichment, and detection, offering a promising platform for rapid airborne pathogen monitoring.

## 1. Introduction

Airborne bacteria often exist as aerosolized particles and are non-uniformly distributed in the environment. The risk of infectious disease is closely associated with both the type and the total concentration of airborne bacteria. Efficient disease control therefore relies on the accurate monitoring and identification of these bacterial species [1,2]. Conventional detection methods—such as plate counting, adenosine triphosphate (ATP) detection, polymerase chain reaction (PCR), and fluorescence detection [3,4,5]—often fall short in enabling rapid identification, largely due to poor integration between bacterial sampling and detection technologies, particularly during the separation and transfer stages. Yan et al. developed a water-soluble filtration membrane that facilitated efficient airborne bacteria capture and elution of airborne bacteria, allowing for quantitative detection via ATP bioluminescence [6]. Bani et al. combined liquid impingement concentration with the loop-mediated isothermal amplification (LAMP) assay, enabling the detection of airborne bacteria within 60 min [7]. However, these approaches still lack seamless integration between sampling and detection and remain susceptible to human intervention. Ko et al. developed an automated bioaerosol monitoring system capable of concentrating airborne bacteria over 10^7^-fold with 99.9% efficiency. This system integrates on-chip culture, imaging, and machine-learning-based detection, completing the entire process within 3 h [8]. Despite its high degree of integration, the system still lacks a rapid identification capability for diverse microbial aerosols. Therefore, developing innovative methods to achieve rapid enrichment, identification, and monitoring of airborne bacteria remains a challenging task [9].

A microfluidic-chip-based platform for research into airborne bacteria has attracted significant interest due to its unique advantages, including microscale fluid manipulation, low reagent consumption, and ease of integration [10,11,12,13,14]. The trapping mechanism and enrichment efficiency of bacteria are largely determined by the morphology and structural configuration of microstructures within the microchannels [15,16,17]. For instance, microfluidic chips with curved or spiral channels can effectively capture target particles via an air-driven flow by leveraging inertial separation and laminar-flow-focusing principles [18]. Designs such as staggered herringbone mixers (SHMs) [19,20] and hybrid double-helix/herringbone structures [21] have achieved exceptional efficiency (≥99.9%) in bacterial capture from aerosols by harnessing inertial centrifugal forces and controlled turbulence. Despite these advances, existing systems still face challenges in achieving sufficiently rapid on-chip microbial capture and enrichment, which remains a critical bottleneck for reliable microbial detection applications. To address this, microchannel arrays [22] and filter membranes [23] have been incorporated into microchips to concentrate airborne bacteria into microchambers at higher flow rates. Additionally, while the hydrophilic nature of the microchannel surface plays an important role in microorganism capture efficiency [19,24], it has been less extensively studied. To enhance bacterial adsorption, we propose modifying the microchannel surface with a coupled polydopamine–chitosan (PDA-co-CS) composite gel. This approach takes full advantage of chitosan’s natural hydrophilicity, excellent biocompatibility, ease of modification, and strong bacterial adsorption capacity [25], combined with the mussel-inspired universal adhesion properties of polydopamine [26]. Thus, microfluidic-chip-based detection methods represent a promising strategy for efficient capture, enrichment, and detection of airborne bacteria.

Surface-enhanced Raman spectroscopy (SERS) has emerged as a powerful analytical technique that synergistically combines the molecular specificity of Raman spectroscopy with plasmon-enhanced signal amplification through interactions between metal nanoparticles and substrate. This approach enables rapid, label-free characterization and identification of airborne bioaerosols with high sensitivity [4,27]. SERS allows for direct bacterial identification without extensive sample pretreatment [28,29,30,31], and its non-destructive nature supports real-time monitoring of bacterial viability in aerosol samples [32]. Particularly in microfluidic applications, SERS stands out as an ideal detection method due to its compatibility with chip-scale integration, facilitated by instrument miniaturization and simplified optical detection requirements [33,34,35,36]. A microfluidic chip integrated with SERS technology has been developed for the separation and real-time detection of airborne bacteria [37]. However, this system was unable to concentrate bacteria on chip, which limited its effectiveness for SERS detection and rendered it unsuitable for applications involving low bacterial concentrations.

In response to the need for efficient enrichment and rapid detection of airborne bacteria, a novel microfluidic biochip integrating a micropillar array with a composite gel interface was designed and fabricated. Leveraging the chaotic vortex flow effect and an enlarged sampling area, the micropillar array enhances bacterial collision and interception efficiency within the microfluidic chip. The surface of the micropillar region was modified with a PDA-co-CS composite gel to improve bacterial capture efficiency. Furthermore, Ag nanostructures were incorporated into the PDA-co-CS composite gel to enable in situ SERS detection of bacteria. The microstructure of the micropillar array coated with the Ag nanostructure @ PDA-co-CS composite gel was characterized using scanning electron microscope. The capture and enrichment conditions of the biochip were optimized, and a detection and identification method for *S. aureus*, *E. coli*, and *Bacillus cereus* in the synthetic aerosols was established based on SERS.

## 2. Materials and Methods

### 2.1. Materials and Reagents

Polydimethylsiloxane (PDMS) was obtained from Dow Corning (Midland, MI, USA). Glutaraldehyde (GA) and 3-aminotriethoxysilane (ATES) were purchased from Aladdin Biochemical Technology Co., Ltd. (Shanghai, China). Chitosan (CS) was supplied by Innochem Technology Co., Ltd. (Beijing, China). Dopamine Hydrochloride (DA•HCl) was obtained from Adamas-beta Technology Co., Ltd. (Shanghai, China). Sybr Green I (SG I, 10,000×) was purchased from Solarbio Science & Technology Co., Ltd. (Beijing, China).

The Tollens reagent was prepared as follows: a 2.5% ammonia solution (Chongqing Chuan Dong Chemical Co., Ltd., Chongqing, China) was added dropwise to 10 mL of 0.05 M silver nitrate solution (Chengdu Kelon Chemical Reagent Factory, Chengdu, China) until the mixture became clear. Then, 5 mL of 0.8 M potassium hydroxide (Chongqing Chuan Dong Chemical Co., Ltd.) and 2.5% ammonia solution were successively added, resulting in the formation of a brown precipitate that subsequently dissolved to yield a transparent and colorless solution. Finally, 1 mL of 0.12 M glucose solution (Shanghai Macklin Biochemical Co., Ltd., Shanghai, China) was added to the mixture, and it was quickly shaken.

*S. aureus* ATCC 6538 and *E. coli* JM109 were kindly provided by Professor Peng of Chongqing Medical University (China). *Bacillus cereus* was supplied by Professor Li of Chongqing University (China). Bacterial strains were cultured in Luria–Bertani medium (Beijing Land Bridge Technology Co., Ltd., Beijing, China) at 37 °C for 12 h. Prior to use, the bacteria were washed with sterile water. Fluorescently labeled *S. aureus* was prepared by adding 100 μL of 5 × SG I to 1 mL of bacterial suspension (10^8^ CFU/mL), followed by incubation in the dark for 10 min.

### 2.2. Preparation of the Microfluidic Biochip

**Figure 1:**
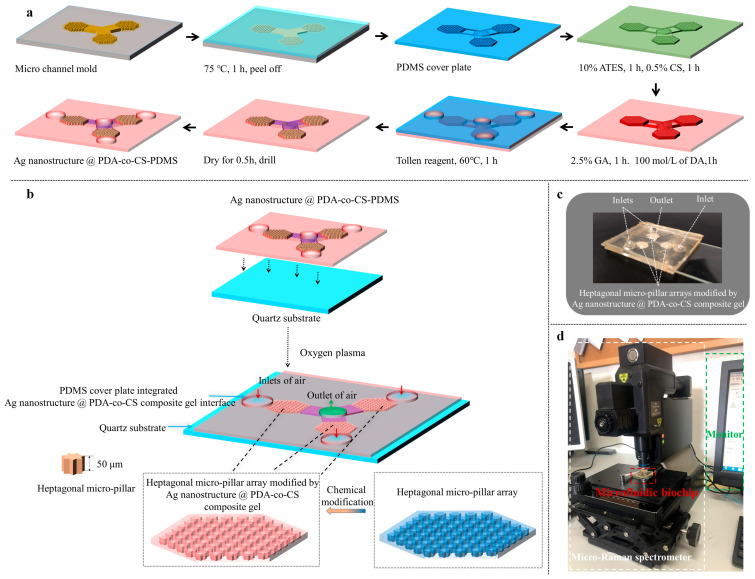
A fabrication schematic of the Ag nanostructure @ PDA-co-CS-PDMS (**a**). A schematic of the designed microfluidic biochip (**b**). A photograph of the microfluidic biochip (**c**). The configuration of the micro-Raman spectrometer system (**d**).

The microchannel mold was fabricated on a silicon substrate via photolithography by Suzhou Wenhao Co., Ltd. (Suzhou, China). The microfluidic biochips were prepared according to the steps illustrated in Figure 1a,b. The PDMS base and curing agent were mixed at a mass ratio of 10:1. The mixture was poured into the mold and cured at 75 °C for 60 min. The resulting PDMS cover plate, featuring heptagonal micropillar arrays and microchannels, was carefully peeled off, treated with oxygen plasma, and immediately immersed in ethanol containing 10% ATES for 1 h. It was then sequentially immersed in 2.5% GA solution, 0.5% CS solution, 2.5% glutaraldehyde solution, and 100 mol L^−1^ of DA•HCl solution, each for 1 h, to form a PDA-co-CS-composite-gel-modified PDMS cover plate.

The modified PDMS cover plate was assembled using a flat PDMS sheet. The flat sheet contained three holes aligned with the micropillar arrays, serving as reaction chambers. Tollens reagent was injected into these chambers and allowed to react at 60 °C for 1 h. After reaction, the unreacted reagent was removed through rinsing with deionized water. The flat PDMS sheet was peeled away, and the substrate was dried for 0.5 h, yielding a PDMS structure modified with the Ag nanostructure @ PDA-co-CS composite gel (Ag nanostructure @ PDA-co-CS-PDMS). Inlet and outlet ports were drilled into the structure. The final biochip was assembled by bonding the Ag nanostructure @ PDA-co-CS-PDMS to a quartz substrate via oxygen plasma treatment (Figure 1b). A physical image of the resulting microfluidic biochip is presented in Figure 1c.

### 2.3. Experimental Setup for Bacterial Enrichment

The experimental setup for bacterial enrichment comprised an aerosol generator (BSW-2A, Beiersi Technology Co., Ltd., Shanghai, China), a sealed gas box (dimensions: 860 × 700 × 520 mm^3^), a micro vacuum pump (KVP04-1.1-24, Kamoer Fluid Tech Co., Ltd., Shanghai, China), a tail gas absorber, and the designed biochip, as shown in Figure S1. The tail gas absorber consisted of a 15 mL centrifuge tube filled with 2 mL of sterile water and 50 μL of sterile vegetable oil, used to collect bacteria that escaped from the biochip. The absorber was connected to the biochip outlet and the vacuum pump via two silicone tubes. One tube below the liquid surface in the absorber, with its opposite end attached to the biochip outlet. The second tube was positioned near the gas outlet of the absorber and connected to the inlet of the micro vacuum pump.

The enrichment procedure was conducted as follows. Bacterial aerosol was generated according to a previously reported method [38]. A total of 10 mL of bacterial suspension was placed into an ultrasonic atomizer (BSW-2A, Beiersi Technology Co., Ltd., Shanghai, China) to produce synthetic aerosol for 5 min. The atomization rate for the bacterial suspension was estimated to be 37% (see Supplementary S1: Test results for atomization rate). Under the negative pressure generated by the vacuum pump, the aerosol was drawn into the biochip, where bacteria were effectively trapped.

### 2.4. Capture and Enrichment of S. aureus in Synthetic Aerosol by the Microfluidic Biochip

Aerosols of *S. aureus* at a concentration of 10^9^ CFU m^−3^ were captured using the microfluidic biochips at flow rates of 0.6, 1.2, and 1.8 L min^−1^ for 20 min each. The number of *S. aureus* cells in the tail gas absorber was quantified using the plate counting method to determine the optimized sampling flow rate.

To optimize the sampling time, aerosol of fluorescently labeled *S. aureus* (10^9^ CFU m^−3^) was generated and enriched for 10, 20, 30, 40, 50, and 60 min, at a flow rate of 1.8 L min^−1^. The captured bacteria were subsequently detected using a fluorescence spectrometer (Omni—λ 300i Monochromator/Spectrograph, Zolix, Beijing, China) and a CX40 upright fluorescence microscope (Ningbo Shunyu Instrument Co., Ltd., Ningbo, China).

The maximum enrichment capacity of the biochip was evaluated using aerosols of fluorescently labeled *S. aureus* at concentrations ranging from 10^4^ to 10^8^ CFU m^−3^. Sampling was performed at 1.8 L min^−1^ for 20 min and detected through fluorescence.

All optimization experiments were conducted in triplicate.

### 2.5. In Situ SERS Detection of S. aureus, E. coli, and Bacillus cereus in Synthetic Aerosols

*S. aureus* aerosols with concentrations ranging from 10^4^ to 10^9^ CFU m^−3^ were continuously introduced into the microfluidic biochip under the optimal conditions. The biochip was placed on the test bench of a micro-Raman spectrometer (Ocean optics, Orlando, FL, USA), as shown in Figure 1d. The system was equipped with a 785 nm laser source and a 40× objective for SERS detection of the captured bacteria. Each sample was measured three times in parallel under the following conditions: a laser power of 11.5 mW and an integration time of 10 s. The average of the acquired SERS spectral data was used for subsequent analysis.

Following the same procedure, capture, enrichment, and SERS detection were performed separately for aerosols of *S. aureus*, *E. coli*, and *Bacillus cereus*. For each bacterial type, thirty replicate samples at a concentration of 10^9^ CFU m^−3^ were analyzed. The corresponding SERS spectra were processed as described above, and a principal component analysis (PCA) was employed to discriminate among the three bacterial species.

### 2.6. Data Processing

The Raman spectra of all samples were subjected to 4-level wavelet decomposition. High-frequency components were removed, and the spectra were reconstructed using low-frequency components. Subsequent smoothing was performed using the Savitzky–Golay method to reduce noise interference further. Baseline correction was then carried out via piecewise polynomial fitting to minimize interference in the analytical model, resulting in baseline-corrected Raman spectra. To mitigate the influence of variations in SERS spectral intensity, all preprocessed spectra were normalized using the MATLAB (Version 9.8) built-in function mapminmax.

For model construction, 20 Raman spectra each of *S. aureus*, *E*. *coli*, and *Bacillus cereus* were randomly selected from the preprocessed dataset to form the training set. An additional 10 spectra from each species, not included in the training set, were used as the prediction set. PCA-based recognition models were developed separately for the three bacterial species using the training set, and the prediction set was employed for blind testing to evaluate the model performance.

## 3. Results

### 3.1. Design of the Microfluidic Biochip

The reported microchip samplers suffer from several limitations when processing low-concentration environmental samples, including low sampling flow rates, limited compatibility with detection methods, and suboptimal efficiency [19,21,22,23]. To address these challenges, a microfluidic biochip integrated with units for sampling, capture, enrichment, and in situ detection was proposed and designed (Figure 1a). The capture region of the biochip was expanded and embedded with the micropillar array. This array was specially designed to provide a high specific surface area and to generate a chaotic vortex flow, thereby increasing bacterial interception sites and enabling efficient aerosol-borne bacterial enrichment. Previous studies have shown that interfaces containing chitosan promote the adsorption of bacteria owing to the readily formed intermolecular interactions between the abundant amino groups in chitosan and the hydroxyl and carboxyl groups present on bacterial surfaces [39]. The adhesion of the chitosan interface to bacteria can be increased after modification with dopamine [38], in which catechol groups play a critical role [40]. Therefore, a coupled polydopamine–chitosan composite gel was constructed on the channel surface of the biochip. Ag nanomaterials have been widely employed in bacterial SERS detection due to their excellent SERS enhancement performance [41]. Therefore, the Ag nanostructure was incorporated into the composite gel to enable in situ SERS detection of captured bacteria. Finally, the microfluidic biochip—integrating a micropillar array and a Ag nanostructure @ PDA-co-CS composite gel interface—was designed for sampling, capture, enrichment, and detection of airborne bacteria.

The microstructure of the micropillar array plays a critical role in the enrichment of airborne bacteria. According to fractal theory, the surface area of the micropillar with some special structures tends to be infinite. As illustrated by theoretical calculations (Figure S2a), there was a clear increment in the lateral surface area of the micropillar with an increased angle number of the micropillar. A larger lateral surface area offers more available sites for bacterial adhesion. The micropillar will trigger a chaotic vortex flow, creating a flow velocity gradient that decreases closer to the pillar surface (Figure S2b). When the micropillar contains angled regions, bacteria entering these areas may become trapped, which had also been validated in previous studies [19]. To improve this capture effect, a heptagonal micropillar design was adopted, providing a greater number of such angled regions. Accordingly, a heptagonal micropillar array was incorporated into the biochip to enhance the bacterial capture efficiency.

The chamber of capture, enrichment, and detection was designed as a regular hexagon with a side length of 3 mm, filled with staggered heptagonal micropillars. The center-to-center distance between adjacent micropillars was 675 μm, and the diameter of each micropillar was 461 μm. Both the height of the micropillars and the depth of the microchannel were uniformly set at 50 μm. To maximize the capture area and enhance the enrichment efficiency, three such hexagonal units were arranged into a ring-shaped array, sharing a common gas outlet (Figure 1b).

### 3.2. Characterization of the Ag Nanostructure @ PDA-co-CS Composite Gel Interface

**Figure 2:**
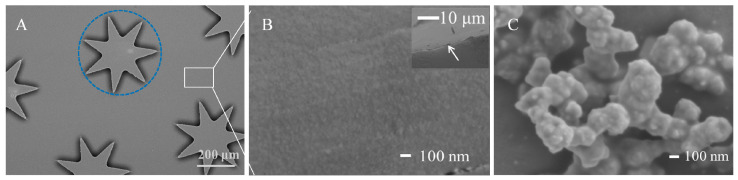
SEM images. (**A**) The PDMS cover plate integrated with the heptagonal micropillar array. The blue circle outlines the top view of a single micropillar. (**B**) A detailed view of the array after modification with a PDA-co-CS composite gel, showing the inter-pillar region indicated by the white rectangle in (**A**). The inset shows a cross-sectional view of the micropillar. The thin gel layer on its top is indicated by the white arrow. (**C**) The Ag nanostructure adhered to the PDA-co-CS composite gel.

The morphology of the heptagonal micropillar array was characterized using scanning electron microscopy (SEM) (Quattros, Thermo Fisher Scientific, Inc., Waltham, MA, USA). As illustrated in Figure 2A, pristine, unmodified heptagonal micropillars are periodically arranged with smooth surfaces, forming the main capture region, in agreement with the design. After modification with the PDA-co-CS composite gel, the surface became uniformly covered with homogeneous nanoscale bulges (Figure 2B). These nanostructures are expected to increase the specific surface area of the micropillar array, thereby enhancing its aerosol capture capability through improved particle–surface interactions. A cross-sectional view of the microchannel in the heptagonal micropillar region is provided in the inset of Figure 2B. The image clearly shows that the tops of the micropillars are covered with a continuous thin gel layer. The uniform coating confirms the successful modification of the microchannel surface with the PDA-co-CS gel. This polymer layer not only serves as an effective adhesion substrate for bacterial capture but also provides a functional foundation for the subsequent integration of silver nanostructures. The efficacy of this surface modification is further confirmed by the altered hydrophilic/hydrophobic properties, as depicted in Figure S3.

Ag nanostructures were deposited within the PDA-co-CS composite gel layer to achieve in situ SERS detection of bacteria. It has been reported that the SERS-active sites form only when the interparticle distance between Ag NPs is less than 10 nm [42]. However, controllably fabricating such well-ordered Ag nanostructures remains challenging. At present, Ag substrates have mainly been prepared through magnetron sputtering [43], electrodeposition [44,45], or chemical reduction [46,47]. For instance, three-dimensional silver nanostructures grown chemically on both sides of layered materials have been utilized for efficient SERS-based bacterial detection [48]. Similarly, the in situ deposition of Ag substrates via a silver mirror reaction has also been effectively applied in SERS detection of *E. coli* and *S. aureus* [47]. In our earlier works, it was proved that Ag nano substrates of various sizes and morphologies could be grown in situ in the micro channel through the silver mirror reaction, and this process was simple and easy to control [49]. Therefore, the silver mirror reaction was chosen to prepare the Ag nano substrate within the composite gel layer.

Silver ions could be completely absorbed into the PDA-co-CS composite gel via its swelling property without dissolving, facilitating in situ growth of the Ag nanostructures. The morphology of the Ag nanostructure was also charactered through SEM. It was shown that Ag nanostructures successfully anchored onto the PDA-co-CS composite gel (Figure 2C). This stable adhesion can be attributed to the abundant amino groups—which become positively charged upon hydrolysis—and catechol functional groups with inherent adhesive properties within the composite gel. The Ag nanostructured substrate exhibited a micro-island morphology resembling coral reefs, containing numerous micro–nano pores that provide ample storage space for bacterial retention. Furthermore, the rough surface of the Ag nanostructure can supply abundant SERS-active sites for bacterial detection.

### 3.3. Capture Efficiency of S. aureus from Synthetic Aerosols Using the Microfluidic Biochip

Synthetic aerosols of *S. aureus* were captured using the microfluidic biochips under different sampling conditions, and the optimal capture conditions were optimized based on the capture efficiency of the microfluidic biochip for *S. aureus* aerosols. The capture efficiency $r_{1}$ was calculated using the following equation. $N_{1}$ is the number of *S. aureus* cells in the tail gas absorber after sampling with the biochip, and $N_{0}$ is the number of *S. aureus* cells directly sucked into the tail gas absorber in the absence of the biochip.

(1) $$r_{1}=\frac{N_{0}-N_{1}}{N_{0}}\times 100\%,$$

The microfluidic biochip was used to capture *S. aureus* aerosols at flow rates of 0.6, 1.2, and 1.8 L min^−1^. After 20 min of sampling, the number of *S. aureus* cells collected in the tail gas absorber was determined using the plate counting method. The capture efficiency was calculated according to Equation (1), and the results are summarized in Table S2. Within the flow range of 0.6 to 1.8 L min^−1^, the capture efficiency for *S. aureus* consistently exceeded 99.9%, which was not affected by the flow rate. This high and stable performance can be attributed to the Ag nanostructure @ PDA-co-CS composite interface, which contains numerous micro–nano pores, combined with the design of the heptagonal micropillar array. Given the low concentration of bacteria in the aerosols, a flow rate of 1.8 L min^−1^ was selected for subsequent experiment to maximize bacterial collection within a practical sampling time.

**Figure 3:**
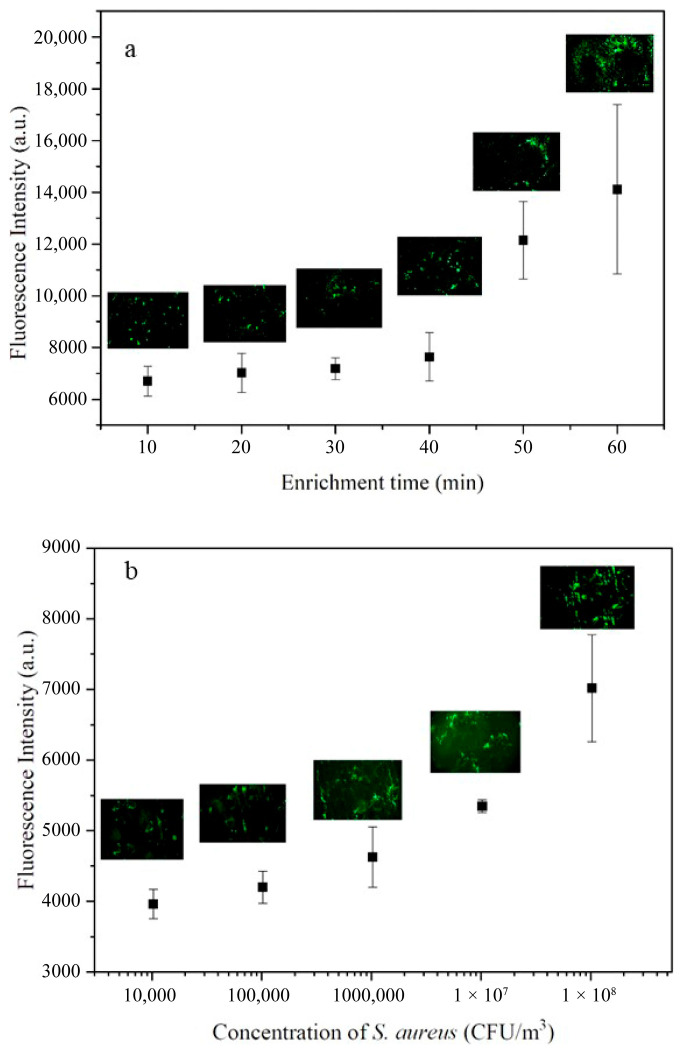
(**a**) Fluorescence intensity changes in *S. aureus* enriched in the biochip with an increase in the enrichment time. (**b**) Fluorescence intensity changes in *S. aureus* enriched in the biochip with an increase in the *S. aureus* concentration in the aerosol. The biochip was integrated with the heptagonal micropillar array and PDA-co-CS composite gel (*n* = 3).

The optimal sampling time and maximum enrichment of the microfluidic biochip for airborne bacteria were optimized. Aerosols containing SYBR Green-labeled *S. aureus* were generated and captured using the biochip integrated with the PDA-co-CS composite gel at a flow rate of 1.8 L min^−1^. The collected *S. aureus* cells within the micropillar array region were detected through fluorescence. It was shown that the amount of enriched *S. aureus* cells was increased with extension of the sampling time (Figure 3a). Under a fixed sampling duration of 20 min, the number of collected *S. aureus* cells substantially increased with the concentration of *S. aureus* cells in the aerosol (Figure 3b). It was confirmed that there were enough space and sites in the biochip to concentrate *S. aureus* across a wide range of aerosol concentrations. For comparison, the synthetic aerosols of *S. aureus* of different concentrations were also tested using natural sedimentation for 20 min, and *S. aureus* was counted after culturing at 37 °C for 24 h. The detectable range of the natural sedimentation method was 1 × 10^4^~1 × 10^7^ CFU m^−3^ (Figure S4). In contrast, the biochip enabled effective capture of *S. aureus* with concentrations both below 1 × 10^4^ CFU m^−3^ and above 1 × 10^7^ CFU m^−3^, where the enriched *S. aureus* could be observed using fluorescence imaging (Figure 3b). In order to enrich enough bacteria for detection and realize rapid detection of the bacterial aerosol, 20 min was selected as the sampling time.

**Figure 4:**
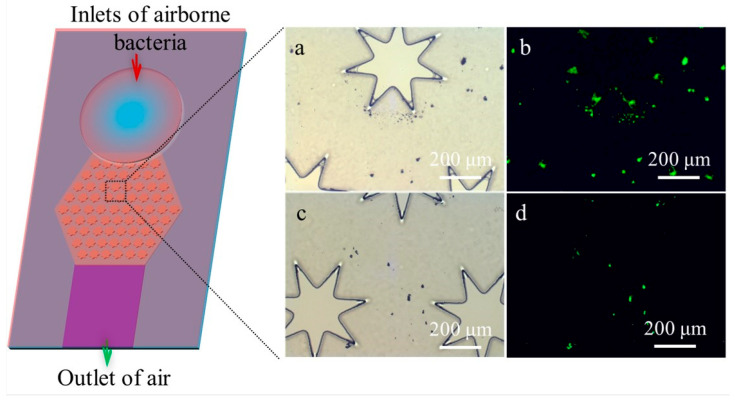
Micrographs of the light field and the fluorescence field after capture of *S. aureus* using a biochip integrated with the PDA-co-CS composite gel (**a**,**b**) and a biochip without modification of the PDA-co-CS composite gel (**c**,**d**).

To verify the adhesion properties of the PDA-co-CS composite gel for bacteria, aerosols of fluorescently labeled *S. aureus* at a concentration of 1 × 10^9^ CFU m^−3^ were captured using the biochip integrated with the PDA-co-CS composite gel. Sampling was performed at a flow rate of 1.8 L min^−1^ for 20 min, and the collected *S. aureus* was observed using a fluorescence microscope. As shown in Figure 4, significantly more *S. aureus* cells were captured around the heptagonal micropillar region in the biochip integrated with the PDA-co-CS composite gel in comparison with the unmodified biochip. Large numbers of *S. aureus* were also observed in the areas adjacent to the heptagonal micropillars. These results demonstrate that the PDA–CS composite gel effectively enhances the capture efficiency for *S. aureus* in the biochip.

To verify the high capture efficiency of the designed biochip, microfluidic biochips with different micro–nano structures were used to capture the aerosol of *S. aureus* under optimized sampling conditions. The number of *S. aureus* cells collected in the tail gas absorber was determined using the plate counting method, and the capture efficiency was calculated using Equation (1). The results are summarized in Table 1. The capture efficiency of *S. aureus* increased from 11.4% to over 99.9% with the integration of the heptagonal micropillar array and the modification of the PDA-co-CS composite gel and the Ag nanostructure. Obviously, the heptagonal micropillar array plays a major role, and the adhesion effect of the PDA-co-CS composite gel and the micro–nano holes of the silver nanostructure also play an important role in improving the efficiency of bacterial capture. It was illustrated that the designed microfluidic biochip efficiently captured *S. aureus* from aerosols due to the integration of the heptagonal micropillar array and the Ag nanostructure @ PDA-co-CS composite gel interface.

### 3.4. In Situ SERS Detection of S. aureus, E. coli, and Bacillus cereus in Synthetic Aerosols

**Figure 5:**
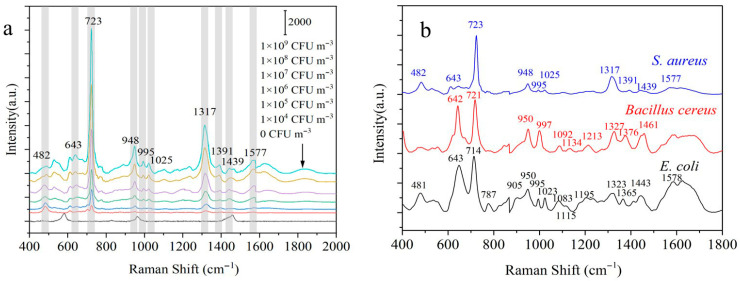
(**a**) SERS spectra of *S. aureus* with gradient concentration. The colored lines correspond to different concentrations, decreasing from top to bottom. The experiment was repeated three times. (**b**) SERS spectra of *S. aureus*, *E. coli*, and *Bacillus cereus*. Twenty groups of samples were measured for each bacterium.

As is known, *S. aureus* is the most common pathogenic bacteria in aerosols, especially those in hospitals, livestock farms, and other places. *S. aureus* was selected as the model for studying the detection performance of the designed biochips. Biochips were used to capture and enrich the *S. aureus* aerosols under the optimal conditions. SERS was applied to detecting the captured bacteria in situ. SERS spectra of *S. aureus* with a concentration of 10^4^~10^9^ CFU m^−3^ were obtained, as shown in Figure 5a. The SERS spectra of *S. aureus* were assigned using research from the literature [29,50,51]. Characteristic Raman peaks were observed at 723 cm^−1^, 948 cm^−1^, and 1317 cm^−1^. The intensity of the characteristic peaks increases with the concentration of *S. aureus*. When the concentration of *S. aureus* was 10^4^ CFU/m^3^, the peaks at 948 cm^−1^ and 1317 cm^−1^ were barely detectable, which was not enough to realize the identification of *S. aureus*. Therefore, the lowest concentration of *S. aureus* that could be detected in situ and identified using the microfluidic biochip was determined to be 10^5^ CFU/m^3^.

Thirty aerosol samples each of *E. coli*, *Bacillus cereus*, and *S. aureus* were analyzed using the microfluidic chip. The obtained SERS spectra were processed through smoothing, baseline correction, and normalization procedures implemented in MATLAB (Version 9.8). From the processed dataset, 20 spectra from each bacterial species were randomly selected to generate the representative average SERS spectra, as shown in Figure 5b. The average spectra revealed characteristic peaks for *E. coli* at 481 cm^−1^, 714 cm^−1^, 787 cm^−1^, 905 cm^−1^, 11,083 cm^−1^, and 1115 cm^−1^ and *Bacillus cereus* at 1092 cm^−1^, 1134 cm^−1^, 1211 cm^−1^, 1327 cm^−1^, 1376 cm^−1^, and 1461 cm^−1^. Since all three bacteria contain many different but identical biological macromolecules, such as lipids, polysaccharides, proteins, and nucleic acids, there were also many similar peaks in the SERS spectra. By comparing the differences in the SERS spectra of *S. aureus*, *E. coli*, and *Bacillus cereus* (Figure S5), the differences in the SERS peaks of different types of bacteria could be more intuitively observed. For example, there were characteristic peaks only for *S. aureus* and *E. coli* at 481/482 cm^−1^, and the peak intensities of the three bacteria were different at 643 cm^−1^. The differences could be used to distinguish the three kinds of bacteria.

**Figure 6:**
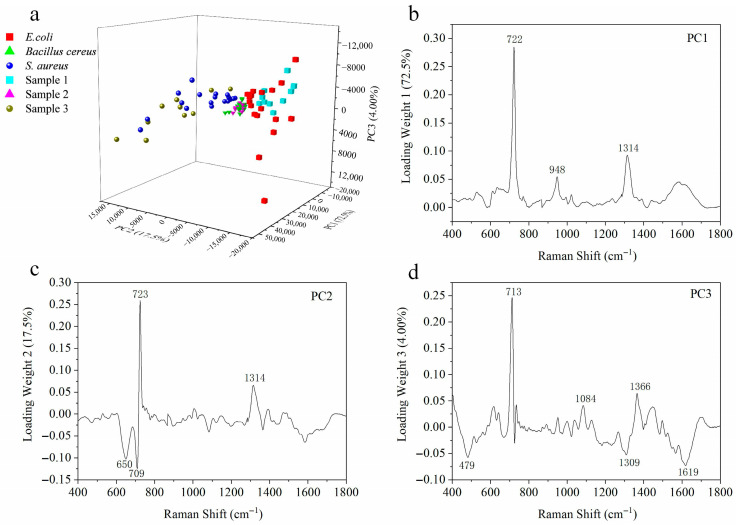
(**a**) The 3D-PCA plot for the three different bacteria and strict class prediction for unknown bacteria samples by the PCA model. (**b**) PC1 loading plot. (**c**) PC2 loading plot. (**d**) PC3 loading plot.

A principal component analysis (PCA) was employed to improve the discrimination of three bacterial species based on their SERS spectral profiles. Spectral data from 60 randomly selected bacterial samples were processed using PCA to extract the principal components, with the first three (PC1, PC2, and PC3) calculated via MATLAB (Version 9.8). These components accounted for 72.5%, 17.5%, and 4.0% of the total variance, respectively, collectively representing 94.0% of the original spectral information, thus capturing most of the relevant data variation. As shown in the combined Figure 6a, the 3D scatter plot illustrates the distribution of all 90 bacterial samples—60 for training and 30 for validation—with each color representing a specific species (*E. coli*, *Bacillus cereus*, or *S. aureus*). The clear clustering of the samples by color demonstrates the effective differentiation among the three species. To validate the method’s predictive capability further, the remaining 30 samples (10 per species) were projected into the same PCA space, achieving a classification accuracy of 100%. These results confirm the reliability and accuracy of the proposed approach to identifying *S. aureus*, *E. coli*, and *Bacillus cereus* and underscore the potential of the biochip and the established method for sampling, capturing, enriching, and efficiently identifying airborne bacteria.

Figure 6b–d present the loading plots of the principal components, in which the characteristic peaks were clearly identified to reveal the molecular mechanism underlying bacterial discrimination. PC1 is predominantly governed by signals at 722 cm^−1^ (phospholipids), 948 cm^−1^ (carbohydrates/polysaccharides), and 1314 cm^−1^ (lipids), reflecting macroscopic differences in cell membrane composition and basal energy metabolism. PC2 is mainly associated with features at 650 cm^−1^ (tyrosine), 709 cm^−1^ (adenine), 723 cm^−1^ (phospholipids), and 1314 cm^−1^ (lipids), indicating that it refines the classification further through coordinated variations in protein composition and nucleic acid content. Meanwhile, PC3 incorporates a broad spectrum of subtle biochemical differences, as evidenced by peaks at 479 cm^−1^ (cell wall polysaccharides), 713 cm^−1^ (adenine), 1084 cm^−1^ (nucleic acid phosphate backbone), 1309 cm^−1^ (lipids), 1366 cm^−1^ (nucleic acids/tryptophan), and 1619 cm^−1^ (aromatic amino acids), thereby serving as a critical fine-tuning factor for achieving perfect classification. These results demonstrate that our SERS-PCA method is not merely a statistical classification tool but also decodes the inherent and systematic biochemical diversity among bacteria in a multi-dimensional and hierarchical manner, providing reliable and interpretable identification outcomes.

## 4. Discussion

This study successfully developed a multifunctional integrated biochip. By leveraging the synergistic effect of physical interception using a heptagonal micropillar array and chemical adsorption by a Ag nanostructure @ polydopamine–co–chitosan (AgNS@PDA-co-CS) composite gel, an exceptionally high capture efficiency for airborne bacteria of up to 99.9% was achieved, effectively addressing the critical challenge of losing target aerosols. Its key innovation lies in the seamless integration of four functional modules—sampling, capture, enrichment, and in situ detection—onto a single microfluidic platform. The AgNS@PDA-co-CS composite gel interface ensured that the captured bacteria were directly located within the SERS hotspots, enabling the rapid and sensitive detection of low-concentration bacterial aerosols (10^5^ CFU m^−3^) within 30 min. The successful discrimination of three bacterial species verifies that the system functions not merely as a sensor but also as an effective classifier based on SERS fingerprint spectra and PCA algorithms, overcoming the limitation of single-target detection.

The microfluidic chips developed for enriching and detecting airborne bacteria, as comprehensively compared in Table 2, demonstrate notable performance improvements over existing platforms. The designed biochip integrates efficient sampling and detection within a single device, enabling rapid monitoring of airborne microorganisms. Its incorporated micropillar array enhances the sample flow rate and processing capacity, while the implementation of SERS detection allows not only for single-species identification but also accurate discrimination of bacterial types when combined with PCA modeling.

This study presents an integrated biochip capable of aerosol microbial sampling, capture, enrichment, and detection. While methodological validation under laboratory conditions has demonstrated the chip’s potential for rapid on-site monitoring, several limitations remain to be addressed in future work. First, the system performance must be evaluated under realistic environmental conditions, including interference from non-biological aerosols such as dust, as well as variations in humidity and temperature. In addition, further optimization of the SERS substrate is essential to enhance the detection sensitivity and reduce the limit of detection. Moving forward, integration and automation are also critical, requiring miniaturized optical components (e.g., lasers, spectrometers) and user-friendly analytical software within a portable platform. Moreover, establishing a reliable quantitative model linking SERS intensity to microbial concentration, as well as improving the biochip’s long-term stability and reusability, will be essential for cost-effectiveness and operational sustainability.

## 5. Conclusions

A biochip integrated with a heptagonal micropillar array and a Ag nanostructure @ PDA-co-CS composite gel interface has been successfully developed for highly efficient airborne bacteria monitoring. The synergistic design achieved a greater than 99.9% capture efficiency and enabled rapid, in situ SERS detection of *S. aureus* at concentrations as low as 10^5^ CFU m^−3^ within 30 min. Furthermore, the platform demonstrated an excellent discriminative capability by successfully distinguishing between *S. aureus*, *E. coli*, and *Bacillus cereus* through SERS fingerprinting combined with PCA. This integrated approach provides a powerful and promising strategy for rapid, multi-target detection of airborne pathogens, offering significant potential for environmental monitoring and public health protection.

## Figures and Tables

**Table 1:** Comparison of capture efficiency of biochip integrated with different micro–nano structures for S. aureus in aerosols (*n* = 3).

Biochip Structures	Amount of *S. aureus* in Tail Gas Absorber (CFU)	Capture Efficiency (%)
Without biochip (blank experiment)	7900 ± 361	-
Without micro–nano structures	7000 ± 629	11.4 ± 4.4
Heptagonal micropillar array	1080 ± 76	86.3 ± 1.4
Heptagonal micropillar array modified with PDA-co-CS composite gel	176 ± 34	97.8 ± 0.5
Heptagonal micropillar array modified with Ag nanostructure @ PDA-co-CS composite gel	0 ± 0	>99.9 ± 0.0

All *S. aureus* cells were captured at a flow rate of 1.8 L min^−1^ for 20 min.

**Table 2:** A comparison of the strengths and weaknesses of our work with other reports.

Function of the Chip	Capture Principles	Capture Efficiency	Flow Rate (L·min^−1^)	Detection Method	Detection Limit	Time (min)	Ref
Enrichment	Horizontal inertial centrifugal force and vertical turbulence	>99.9%	0.004	-	-	20	[21]
Detection	-	-	-	A silicon nanowire field-effect-transistor biosensor	4 × 10^4^ particles mL^−1^	>10	[52]
Detection	-	97.9% (cyclone air sampler)	-	SPR/LSPR ^1^	297.3 TCID_50_/mL_buffer_	~30	[53]
Detection	-	-	-	Electrochemical method	<600 CFU m^−3^	~70	[54]
Separation, detection	Inertial separation	-	0.014	AC impedance sensor	1 × 10^3^ CFU mL^−1^	<20	[55]
Focus, separation, detection	Dual-sheath flow-focusing	-	0.012	Dynamic transmission spectroscopy analysis, single-photon measurement	-	-	[56]
Enrichment, detection	Filtration	>99%	0.071	LAMP ^2^	10^9^ CFU m^−3^	>60	[23]
Enrichment, detection	Chaotic vortex flow	-	0.004	LAMP	-	70	[57]
Enrichment, detection	Chaotic vortex flow	-	-	LAMP	~300 CFU m^−3^	180	[58]
Enrichment, in-situ detection	Chaotic vortex flow and adsorption of Ag nanostructure @ PDA-co-CS composite gel	>99.9%	1.8	SERS ^3^	10^5^ CFU m^−3^	≤25	This work

SPR is short for Surface Plasmon Resonance, and LSPR is short for Localized Surface Plasmon Resonance; LAMP is short for loop-mediated isothermal amplification; SERS is the abbreviation of surface-enhanced Raman spectroscopy.

## Data Availability

The original contributions presented in this study are included in the article/Supplementary Materials.

## Supplementary Materials

The following supporting information can be downloaded at https://www.mdpi.com/article/10.3390/bios15110720/s1. Figure S1: A picture of the real product and the experimental assembly for bacterial enrichment. Figure S2: (a) A theoretical calculation formula for micropillar circumference and lateral area, where *a* is the side length of one corner of the micropillar, *Lm* is the bottom perimeter of the micropillar, and *Sm* is the lateral area of the micropillar. (b) The simulation results on the vortex flow produced by the heptagonal micropillar (the flow rates were set to 143 m/s). Figure S3: The test results on the contact angle. (a) PDMS sheet. (b) PDMS sheet modified with chitosan gel. (c) PDMS sheet modified with PDA-co-CS composite gel. Figure S4: Test results for synthetic aerosols of *S. aureus* of different concentrations using the natural sedimentation method (*n* = 3). Figure S5: (a) Difference in spectra between *E. coli* and *Bacillus cereus*, (b) difference in spectra between *S. aureus* and *E. coli*, (c) difference in spectra between *S. aureus* and *Bacillus cereus*. Figure S6: SEM images of *S. aureus*, *E. coli,* and *Bacillus cereus.* Table S1: Test results for atomization rate. Table S2: Capture efficiency for *S. aureus* using the biochips under different flow rates (*n* = 3).
