# Supplementary material for: Microfluidic Biochip Integrated with Composite Gel Composed of Silver Nanostructure @ Polydopamine–co–Chitosan for Rapid Detection of Airborne Bacteria
Source: Biosensors (Basel). 2025 Oct 30;15(11):720. doi: 10.3390/bios15110720 (PMC12650106; doi:10.3390/bios15110720)
Supplement: Supplementary file 1 [file biosensors-15-00720-s001.zip › biosensors-3879740-supplementary.pdf]

Supplementary Information for

Microfluidic Biochip Integrated with Composite Gel Composed of Silver Nanostructure @ Polydopamine–co–Chitosan for Rapid Detection of Airborne Bacteria

Xi Su [1,2], Xinyu He [2,3], Chuang Ge [4], Yipei Wang [2,5] and Yi Xu [2,3,*]

1 School of Advanced Materials Engineering, Jiaxing Nanhu University, Jiaxing 314001, China;
2 Key Disciplines Lab of Novel Micro-Nano Devices and System Technology, Key Laboratory of Optoelectronic Technology and Systems, Ministry of Education, Chongqing University, Chongqing 400044, China; China;
3 College of Chemistry and Chemical Engineering, Chongqing University of Science and Technology, Chongqing 401331, China
4 Key Laboratory of Translational Research for Cancer Metastasis and Individualized Treatment, Chongqing University Cancer Hospital, Chongqing 400030, China;
5 School of Optoelectronic Engineering, Chongqing University, Shapingba, Chongqing 400044, China
* Correspondence: xuyibbd@cqu.edu.cn

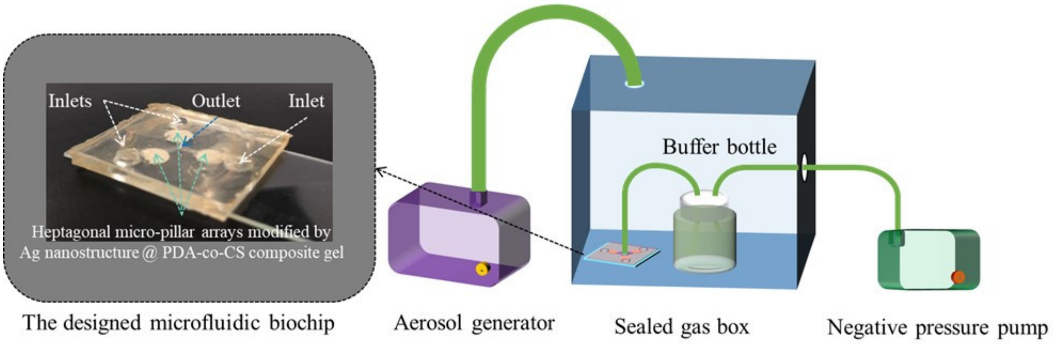

Figure S1: A picture of the real product and the experimental assembly for bacterial enrichment.

S1. Analysis of the atomization rate

The aerosol was prepared according to our previously reported method [1]. A total fo 10 mL of the bacterial suspension was added into an ultrasonic atomizer (BSW-2A, Beiersi Technology Co., LTD., Shanghai, China) to generate the synthetic aerosol for 5 min. The pipeline connecting the atomizer and the sealed gas box was flushed with sterile water after atomization. The eluent was mixed with the remaining bacterial suspension, and the

volume of the mixture was fixed to 10 mL. The number of bacteria in the suspension was determined using the plate counting method before and after atomization.

The atomization rate f_1 was calculated using the following formula:

$$f_1 = \frac{N_{s0} - N_{s1}}{N_{s0}} \times 100\%$$

where N_{s0} is the number of bacteria in the suspension before atomization, and N_{s1} is the number of bacteria in the suspension after atomization. The calculation results for atomization rate are shown in Table S1: the atomization rate is 31.7±0.2 % according to the results of three experiments. This means that the number of bacteria in the artificial aerosols accounted for about 37% of the total bacteria in the suspension.

Table S1: Test results for atomization rate (n=3).

No.	N_{s0} (CFU)	N_{s1} (CFU)	f_1(%)	
1	6.7×10^7	4.6×10^7	31.6	
2	4.9×10^7	3.4×10^7	31.6	31.7±0.2
3	5.3×10^7	3.6×10^7	31.9	

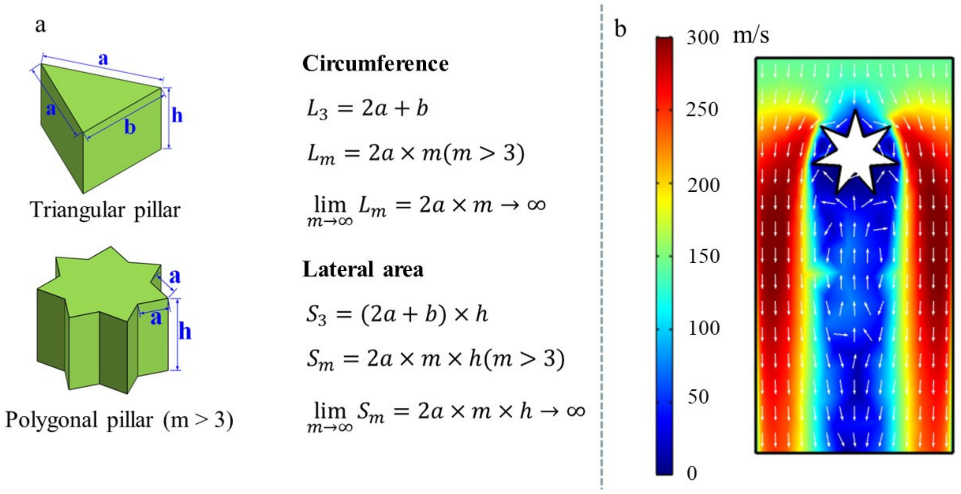

Figure S2: (a) A theoretical calculation formula for micropillar circumference and lateral area, where *a* is the side length of one corner of the micropillar, *Lm* is the bottom perimeter of the micropillar, and *Sm* is the lateral area of the micro-pillar. (b) The simulation results on the vortex flow produced by the heptagonal micropillar (the flow rates were set to 143 m/s).

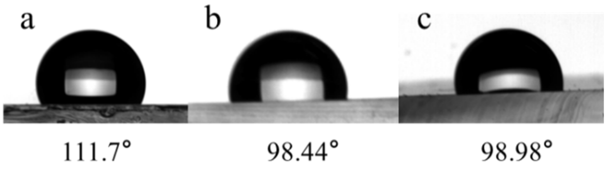

Figure S3: The test results on the contact angle**.** (a) PDMS sheet. (b) PDMS sheet modified with chitosan gel. (c) PDMS sheet modified with PDA-co-CS composite gel.

Table S2: Capture efficiency for *S. aureus* using the biochips under different flow rates (n=3).

Flow rate (L/min)	Amount of *S. aureus* in tail gas absorber (CFU)			Capture efficiency (%)	
	Without biochip	Biochip[1]	The designed biochip[2]	Biochip[1]	The designed biochip[2]
0.6	4265±7	945±19	0	77.8	>99.9
1.2	5650±11	1051±15	0	81.4	>99.9
1.8	7900±7	176±4	0	97.8	>99.9

([1]Biochip integrated with heptagonal micropillar arrays and PDA-co-CS composite gel. [2] Biochip integrated with heptagonal micropillar arrays and Ag nanostructure @ PDA-co-CS composite gel.)

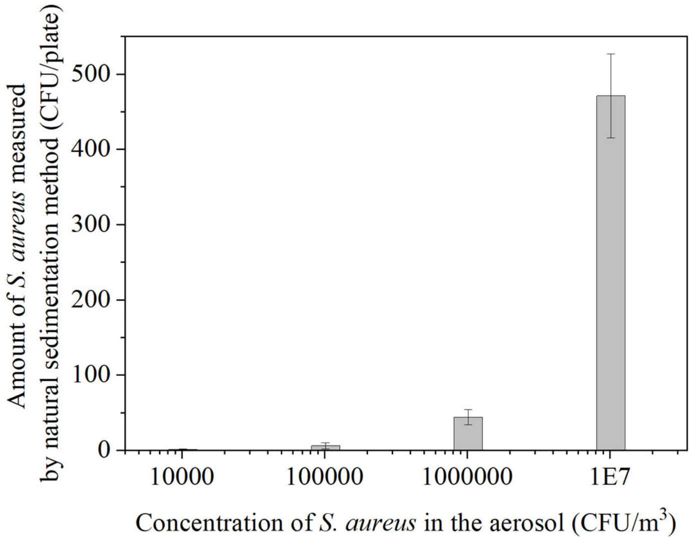

Figure S4: Test results for synthetic aerosols of *S. aureus* of different concentrations using the natural sedimentation method (n=3).

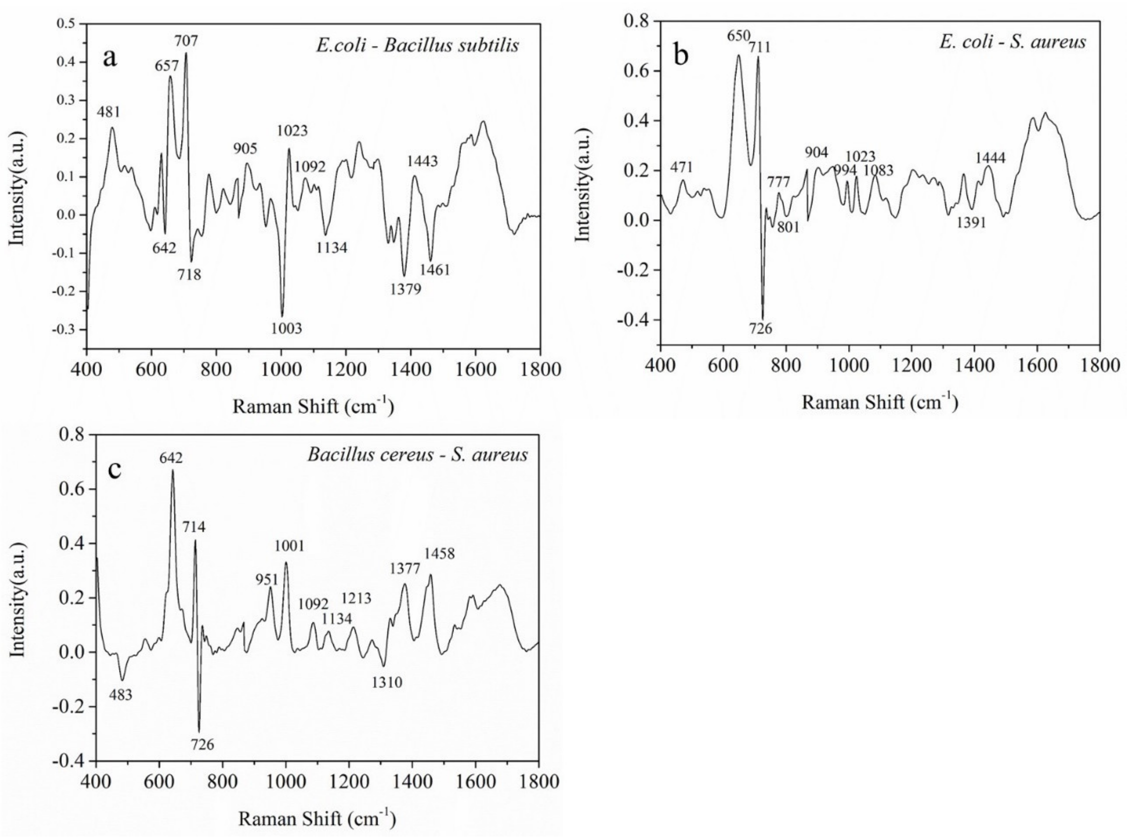

Figure S5: (a) Difference in spectra between *E. coli* and *Bacillus cereus*, (b) difference in spectra between *S. aureus* and *E. coli*, (c) difference in spectra between *S. aureus* and *Bacillus cereus*

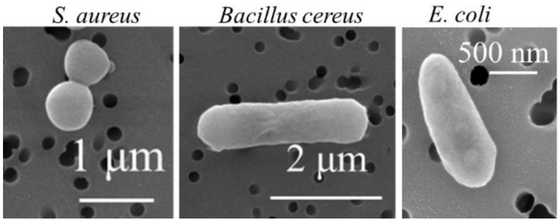

Figure S6: SEM images of *S. aureus*, *E. coli*, and *Bacillus cereus.*

As shown in Fig S6, *Staphylococcus aureus* exhibits a spherical morphology with an average diameter of approximately 0.8 μm. In contrast, both *Bacillus cereus* and *Escherichia coli* display rod-shaped forms, with the former measuring about 0.5 μm in width and 1.2 μm in length and the latter approximately 0.7 μm in width and 1.2 μm in length.

Reference

1 Su, X.; Ren, R.; Wu, Y.; Li, S.; Ge, C.; Liu, L.; Xu, Y., Study of biochip integrated with microelectrodes modified by poly-dopamine-co-chitosan composite gel for separation, enrichment and detection of microbes in the aerosol. *Biosensors and*

Bioelectronics **2021,** *176*, 112931. https://doi.org/10.1016/j.bios.2020.112931.
